# An inverse association between serum soluble receptor of advanced glycation end products and hyperandrogenism and potential implication in polycystic ovary syndrome patients

**DOI:** 10.1186/s12958-017-0227-8

**Published:** 2017-01-26

**Authors:** Yu Liao, Rong Huang, Yun Sun, Jiang Yue, Jun Zheng, Lihua Wang, Tao Tao, Jing Ma, Shengxian Li, Wei Liu

**Affiliations:** 1 Department of Endocrinology and Metabolism, South Campus, Ren Ji Hospital, School of Medicine, Shanghai Jiao Tong University, Shanghai, China; 2 Department of Endocrinology and Metabolism, Ren Ji Hospital, School of Medicine, Shanghai Jiao Tong University, Shanghai, China; 3 Shanghai Key laboratory for Assisted Reproduction and Reproductive Genetics, Center for Reproductive Medicine, Ren Ji Hospital, School of Medicine, Shanghai Jiao Tong University, Shanghai, China

**Keywords:** PCOS, AGEs, sRAGE, Obesity, Insulin resistance, Hyperandrogenism

## Abstract

**Background:**

Studies found that AGE-RAGE system is closely related to insulin resistance and hyperandrogenemia, which are two core pathophysiological processes in polycystic ovary syndrome (PCOS). This study is to investigate the relationship among advanced glycation end-products/soluble receptor of advanced glycation end-products (AGEs/sRAGE) and anthropometric evaluation, homeostatic model assessment-insulin resistance (HOMA-IR), free androgen index (FAI) in reproductive-aged PCOS patients.

**Methods:**

One hundred and forty-eight Chinese women with PCOS were enrolled in this study. Subgroups were divided according to body mass index (BMI), waist circumference (WC), quartile intervals of HOMA-IR and androgen levels. The relationships between AGEs/sRAGE and above clinical markers were assessed by Pearson’s correlation analyses.

**Results:**

Serum AGEs showed a gradually increased tendency with BMI and WC. It reached statistical significant between the normal weight group (BMI < 24 kg/m^2^) and the obesity group (BMI ≥ 28 kg/m^2^) . The sRAGE levels gradually decreased with increasing BMI, WC, HOMA-IR and FAI respectively. Furthermore, the differences between each group were statistical significant. The correlation analysis showed a positive correlation between BMI and serum AGEs levels. On the contrary, the sRAGE levels showed significantly inverse correlations with BMI, WC, HOMA-IR and FAI. The optimal point of sRAGE for the presence of insulin resistance was 704.097 pg/ml by ROC curve analysis.

**Conclusions:**

Along with the body fat accumulation, the serum levels of AGEs were increased, whereas, the serum levels of sRAGE were reduced in obese PCOS patients. The serum levels of sRAGE, which is a decoy receptor, dramatically decreased in the patients with more severe insulin resistant states and higher FAI, which might be a potential biomarker and a promising therapeutic target in the treatment of PCOS, especially in obese subjects.

## Background

Polycystic ovary syndrome (PCOS) is the most common endocrinological disorder in women of reproductive age [1], which is characterized by hyperandrogenism, chronic anovulation and/or PCO morphology, with the exclusion of the adrenal, ovary and pituitary disorders [2]. PCOS is also characterized by metabolic aberrations, such as hyperinsulinemia, insulin resistance (IR), impaired glucose tolerance (IGT), diabetes mellitus (DM) and increased several cardiovascular risk factors [3, 4]. However, the etiology of PCOS remains largely unknown. Advanced glycation end products (AGEs) are the end products of a chemical procedure called Maillard reaction [5], which are formed by nonenzymatic modification of proteins, lipids, and nucleic acids [6]. Serum AGEs have been shown to be elevated in women with PCOS, compared with controls. In recent years, AGEs have been shown to be implicated in the pathogenesis of PCOS [7–9]. In addition, they also have been proposed to be among the main intermediaries of several diseases, such as type 2 diabetes mellitus (T2DM), obesity, metabolic syndrome, aging and inflammation [9, 10].

The action of AGEs is classified as receptor independent (by binding to the extracellular matrix) and receptor dependent (by binding to a cellular receptor called RAGE) manners [6]. The AGE-RAGE interaction leads to activation of signaling pathways that in turn activate nuclear factor-kappa B(NF-κB), promoting the development of a proinflammatory state, cellular toxicity and damage [11]. An extracellular form of RAGE circulates in the blood and follicular fluid is called soluble receptor of AGEs (sRAGE). In contrast to RAGE, sRAGE acts as a decoy receptor, as it binds circulating AGEs and prevents the adverse intracellular events of the AGE-RAGE binding [11, 12]. Therefore, sRAGE is often considered as a ‘good’ receptor since its levels have shown to be down-regulated in hyperglycemia and obesity [13, 14]. However, there were very few studies of the variant levels of serum AGEs and sRAGE in PCOS patients with different body mass index (BMI), waist circumference (WC), homeostatic model assessment-insulin resistance (HOMA-IR), total testosterone (TT) and free androgen index (FAI).

The aim of this study is to investigate the variations of AGEs/sRAGE levels in Chinese Han population with PCOS. The relations between their levels and clinical marks, BMI, WC, HOMA-IR, TT and FAI, have also been studied. Finally, the cut-off values of sRAGE at insulin resistance risk were determined, as well.

## Methods

### Subjects

One hundred and forty-eight Chinese women with PCOS between the age of 18 and 44 years were enrolled in this study. They were recruited from outpatient of the Department of Endocrinology in Ren Ji Hospital, School of Medicine, Shanghai Jiao Tong University from 2014 to 2015. PCOS was diagnosed using the Rotterdam criteria [15], which includes the presence of at least two of the following three components: (i) oligo- and/or anovulation: fewer than eight spontaneous hemorrhagic episodes/year, (ii) clinical and/or biochemical signs of hyperandrogenism, (iii) polycystic ovaries on ultrasonography: at least 12 small follicles in at least one ovary and/or ovarian volume >10 mL, and exclusion of related disorders. All subjects were selected to be nonsmokers, and patients with clinical thyroid dysfunction and taking medication known to affect carbohydrates, lipids or hormones were excluded from the study.

### Methods

Clinical data including age, height, weight, BMI and WC were collected. All subjects underwent anthropometric evaluation. Height was measured using a digital stadiometer. Weight was measured using a calibrated balance scale. BMI was calculated as the weight in kilograms divided by the height in meters squared. WC was measured at the highest point of the iliac crest. Fasting blood samples were drawn at 8 o’clock during day 2–5 of the menstrual cycle. If the patient had amenorrhoea for more than 3 months, the examination was performed randomly. Blood samples were centrifuged, aliquoted and immediately frozen at −80 °C for biochemical analysis. The fasting plasma glucose level was quantified by the glucose oxidase method, and serum insulin was measured by radioimmunoassay. The levels of serum hormones, including TT, sex-hormone-binding globulin (SHBG) were detected by chemiluminescence (Elecsys autoanalyzer, Roche Diagnostics, Mannheim, Germany). HOMA-IR was determined as fasting glucose (mmol/L) × fasting insulin (mU/L) / 22.5. Insulin resistance state was defined as HOMA-IR above 2.29 for the ROC analysis [16]. According to the quartile intervals of HOMA-IR levels, women with PCOS were divided into four groups as follows: H1 (<2.49), H2 (2.49–3.92), H3 (3.92–5.88) and H4 (≥5.88). FAI was calculated as TT (nmol/L) × 100/SHBG (nmol/L). According to the quartile intervals of FAI levels, four groups were divided as follows: F1 (<3.41), F2 (3.41–6.73), F3 (6.73–10.59) and F4 (≥10.59). According to the quartile intervals of TT levels, four groups were: T1 (<1.22 nmol/L), T2 (1.22–2.09 nmol/L), T3 (2.09–2.69 nmol/L) and T4 (≥2.69 nmol/L). Serum AGEs and sRAGE levels were measured in duplicate by enzyme-linked immunosorbent assay (ELISA) (AGEs ELISA Kit, Cellbiolabs, INC, USA, sRAGE ELISA Kit, Enzo Life Sciences, USA) according to the manufacturer’s protocol.

### Statistical analysis

Statistical analysis was performed using the SPSS program (version 16.0) (SPSS Inc., Chicago, IL, USA). Statistically significance was accepted at *P* < 0.05. Data was presented as mean ± standard error (mean ± SE). Kolmogorov–Smirnov test was performed to assess the normal distribution. Comparisons among each group were performed using *t*-test or one-way analysis of variance (ANOVA) with the Sidak method. Pearson’s correlation was used to assess the relationship between two variables. To determine the optimal thresholds, the point on the ROC curve with maximum Youden index [sensitivity-(1-specificity)] was calculated.

## Results

1. **Anthropometric characteristics, metabolic and hormonal profile of enrolled PCOS patients**
  The clinical characteristics of the whole 148 patients are summarized in Table 1.

**Table 1:** Anthropometric characteristics, metabolic and hormonal profile of enrolled PCOS patients

Number	148
Age (years)	26.000 ± 0.449
BMI (kg/m^2^)	25.837 ± 0.423
WC (cm)	86.576 ± 1.087
FPG (mmol/L)	5.031 ± 0.083
FINS (μIU/ml)	21.278 ± 1.109
HOMA-IR	4.980 ± 0.345
TT (nmol/L)	1.981 ± 0.071
SHBG (nmol/L)	38.463 ± 2.781
FAI	8.338 ± 0.567

Data are mean ± SE. *BMI* body mass index, *WC* waist circumference, *FPG* fasting plasma glucose, *FINS* fasting insulin, *HOMA-IR* homeostatic model assessment-insulin resistance, *TT* total testosterone, *SHBG* sex-hormone-binding globulin, *FAI* free androgen index
2. **The levels of serum AGEs and sRAGE in normal weight, overweight and obese PCOS patients**
  The patients were divided into three groups on the basis of BMI according to the Chinese adult overweight and obesity prevention and control guidelines: the normal weight group [NW, BMI <24 kg/m^2^, *n* = 53], the overweight group (OW, 24≤ BMI <28 kg/m^2^, *n* = 50) and the obesity group (OB, BMI ≥28 kg/m^2^, *n* = 45). As shown in Fig. 1a, the concentrations of AGEs showed a gradually increased tendency in all three groups and a statistically significant difference was noted between the normal weight group and the obesity group (*P* = 0.009, after adjustment for FPG, *P* = 0.029). Meanwhile, a statistically significant difference was also noted between the overweight group and the obesity group (*P* = 0.018, after adjustment for FPG, *P* = 0.055). As shown in Fig. 1b, the sRAGE concentrations were significantly higher in the normal weight group than that in the obesity group (*P* < 0.001, after adjustment for FPG, *P* < 0.001). Similarly, the concentrations were also significantly higher in the overweight group than in the obesity group (*P* = 0.003, after adjustment for FPG, *P* = 0.014).

**Fig. 1:**
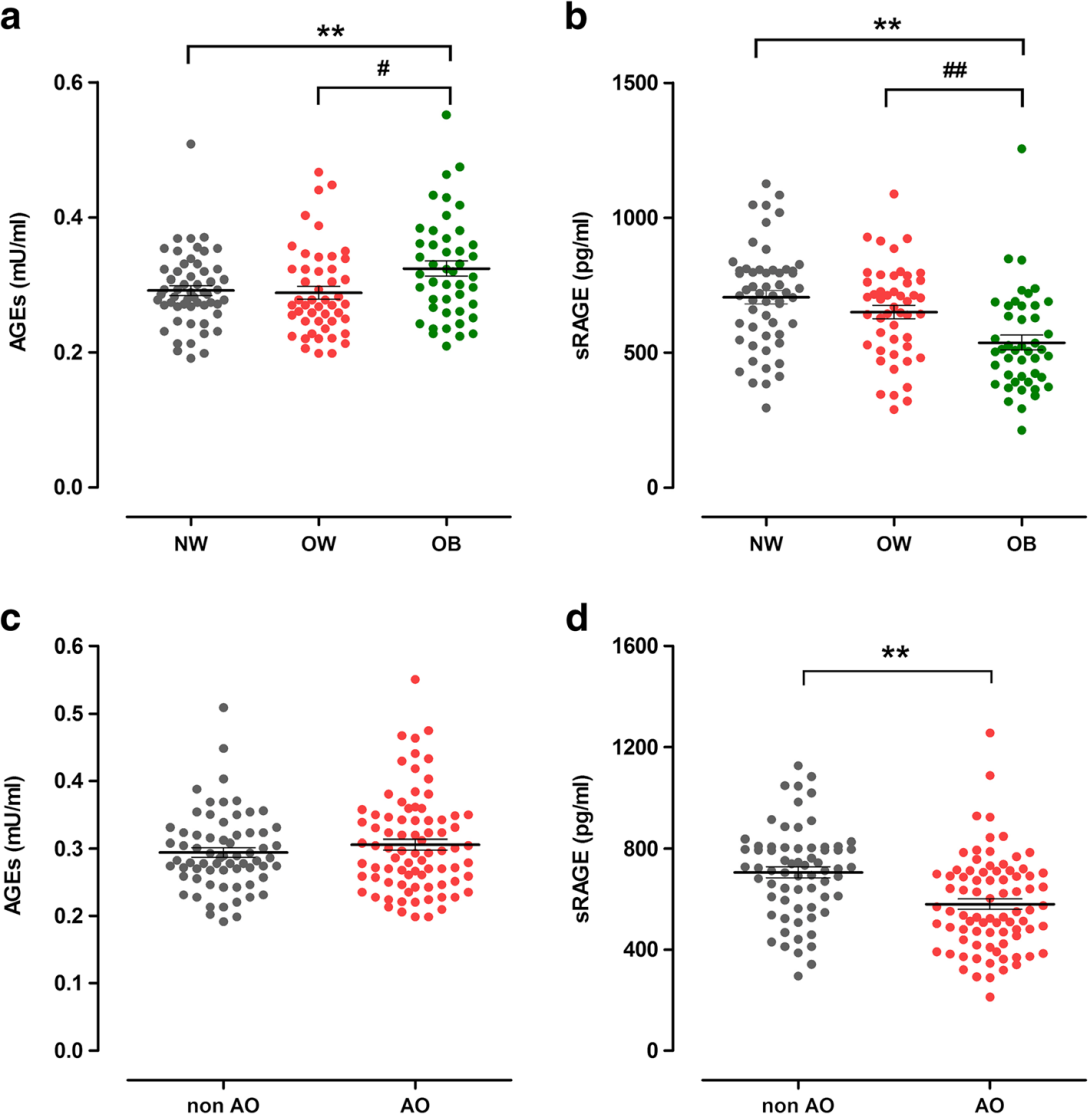
The levels of AGEs and sRAGE in different groups were divided according to anthropometric parameters. Data are mean ± SE. AGEs, advanced glycation end products. sRAGE, soluble receptor of AGEs. BMI, body mass index. WC, waist circumference. **a** and **b** represent the levels of serum AGEs and sRAGE in different groups were divided according to BMI. NW, the normal weight group (BMI <24 kg/m^2^, *n* = 53). OW, the overweight group (24 ≤ BMI < 28 kg/m^2^, *n* = 50). OB, the obesity group (BMI ≥ 28 kg/m^2^, *n* = 45). P value for analysis of variance for differences between groups: * < 0.05 compared with NW, ** < 0.01 compared with NW. # < 0.05 compared with OW, ## < 0.01 compared with OW. **c** and **d** represent the levels of serum AGEs and sRAGE in different groups were divided according to WC. non AO, non-abdominal obese group (WC ≤85 cm, *n* = 67). OA, abdominal obese group (WC >85 cm, *n* = 81). *P* value for analysis of variance for differences between groups: * < 0.05, ** < 0.01
3. **The levels of serum AGEs and sRAGE in abdominal obese and non-abdominal obese PCOS patients**
  According to the abdominal obesity diagnosis standard in China, we divided the patients into two groups: the non-abdominal obese group (non AO, WC ≤85 cm, *n* = 67) and the abdominal obese group (AO, WC >85 cm, *n* = 81). As shown in Fig. 1c, d, the AGEs concentrations were positive correlated with the WC. However, there was no statistical significance. On the contrary, a statistically significant difference was noted in the serum sRAGE concentrations between these two groups (*P* < 0.001).
4. **The levels of serum AGEs and sRAGE in groups with variant HOMA-IR levels**
  As shown in Fig. 2a, b, the AGEs concentrations were not correlated with increased HOMA-IR values. However, the sRAGE concentrations showed a gradually decreased tendency and reached statistical significances from H1 to H4 (*P* = 0.003), after adjustment for age, the four groups remained reaching statistical significance (*P* = 0.004). Among them, the sRAGE concentrations in group H1 were significantly higher than group H3 (*P* = 0.001, after adjustment for age, *P* = 0.008) and H4 (*P* = 0.001, after adjustment for age, *P* = 0.011).

**Fig. 2:**
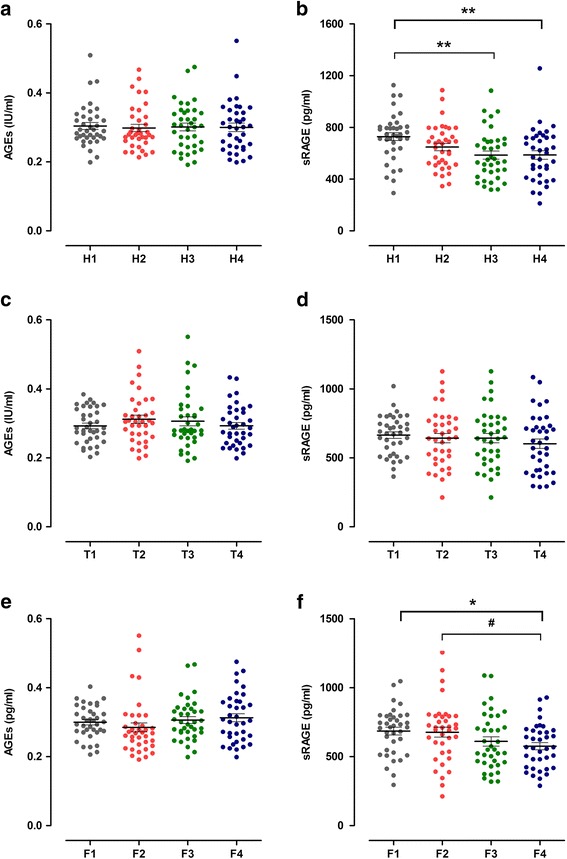
Results of ELISA for AGEs and sRAGE in the quartile intervals groups. Data are mean ± SE. AGEs, advanced glycation end products. sRAGE, soluble receptor of AGEs. HOMA-IR, homeostatic model assessment-insulin resistance. TT, total testosterone. FAI, free androgen index. **a** and **b** represent the levels of serum AGEs and sRAGE in the four HOMA-IR groups. H1 (HOMA-IR <2.49, *n* = 37), H2 (2.49 ≤ HOMA-IR < 3.92, *n* = 37), H2 (3.92 ≤ HOMA-IR < 5.88, n = 37), H4 (HOMA-IR ≥ 5.88, *n* = 37). P value for analysis of variance for differences between groups: * < 0.05 compared with H1, ** < 0.01 compared with H1. **c** and **d** represent the levels of serum AGEs and sRAGE in the four TT groups. T1 (TT < 1.22 nmol/L, *n* = 37), T2 (1.22 nmol/L ≤ TT < 2.09 nmol/L, *n* = 37), T3 (2.09 nmol/L ≤ TT < 2.69 nmol/L, *n* = 37), T4 (TT ≥ 2.69 nmol/L, *n* = 37). **e** and **f** represent the levels of serum AGEs and sRAGE in the four FAI groups. F1 (FAI < 3.41, *n* = 37), F2 (3.41 ≤ FAI < 6.73, *n* = 37), F3 (6.73 ≤ FAI < 10.59, *n* = 37), F4 (FAI ≥ 10.59, *n* = 37). *P* value for analysis of variance for differences between groups: * < 0.05 compared with F1. # < 0.05 compared with F2
5. **The serum levels of AGEs and sRAGE in groups with variant TT levels**
  As shown in Fig. 2c, d, there were no differences of AGEs concentrations among each group with variant TT levels. In contrast, the sRAGE concentrations showed a gradually decreased tendency, but there were no significant differences among each group.
6. **The levels of serum AGEs and sRAGE in groups with variant FAI levels**
  As shown in Fig. 2e, f, all four groups with variant FAI values were not differ in the AGEs concentrations. The sRAGE concentration was inversely correlated with FAI value (*P* = 0.038). And the sRAGE concentrations in group F4 were significantly lower than group F1 (*P* = 0.014) and F2 (*P* = 0.022).
7. **Correlations between the AGEs or sRAGE concentrations and other endocrinological parameters**
  As presented in Fig. 3, there was a positive correlation between BMI and serum AGEs levels. On the contrary, the serum sRAGE levels showed a significantly inverse correlation with BMI, WC, HOMA-IR and FAI. After adjustment for age and/or FPG, the correlation was still observed. In addition, we carried on the multivariate analysis and found that BMI was the independent contribution for its association with sRAGE (*P* = 0.000).

**Fig. 3:**
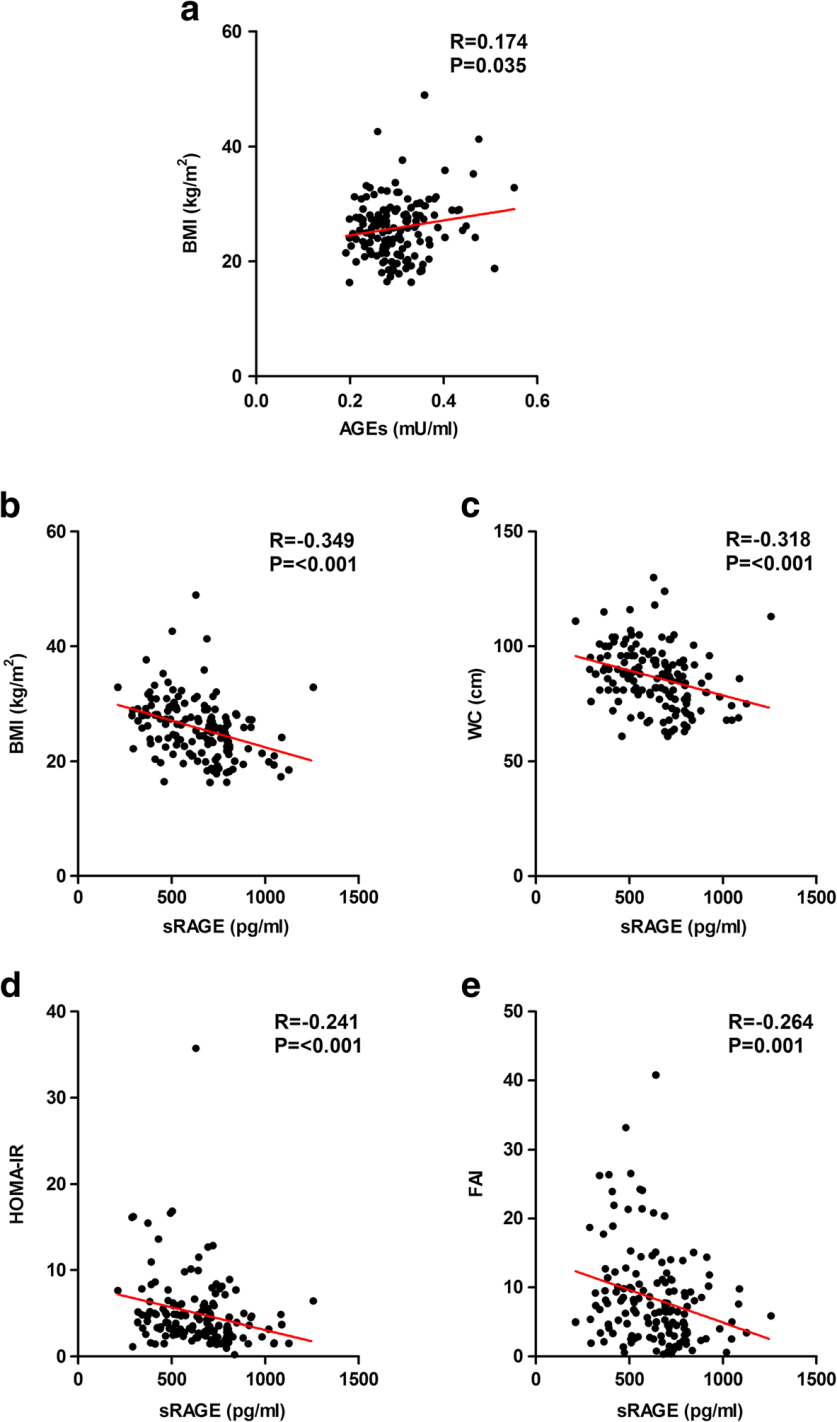
Correlations between AGEs/sRAGE and other endocrinological parameters. AGEs, advanced glycation end products. sRAGE, soluble receptor of AGEs. BMI, body mass index. WC, waist circumference. HOMA-IR, homeostatic model assessment-insulin resistance. FAI, free androgen index. **a** represents the correlation between the AGEs and BMI. **b**, **c**, **d**, **e** represent the correlation between sRAGE and BMI, WC, HOMA-IR and FAI respectively
8. **The cut-off point of sRAGE determined by ROC analysis**
  In the total participants’ pool, the cut-off 704.097 pg/ml for sRAGE was the best threshold for insulin resistance valued by HOMA-IR [16]. It maximized the Youden index (sensitivity = 77.4%, specificity = 29.1%, Youden index = 0.483). The area under the curves in the ROC analysis was 0.717 (Fig. 4).

**Fig. 4:**
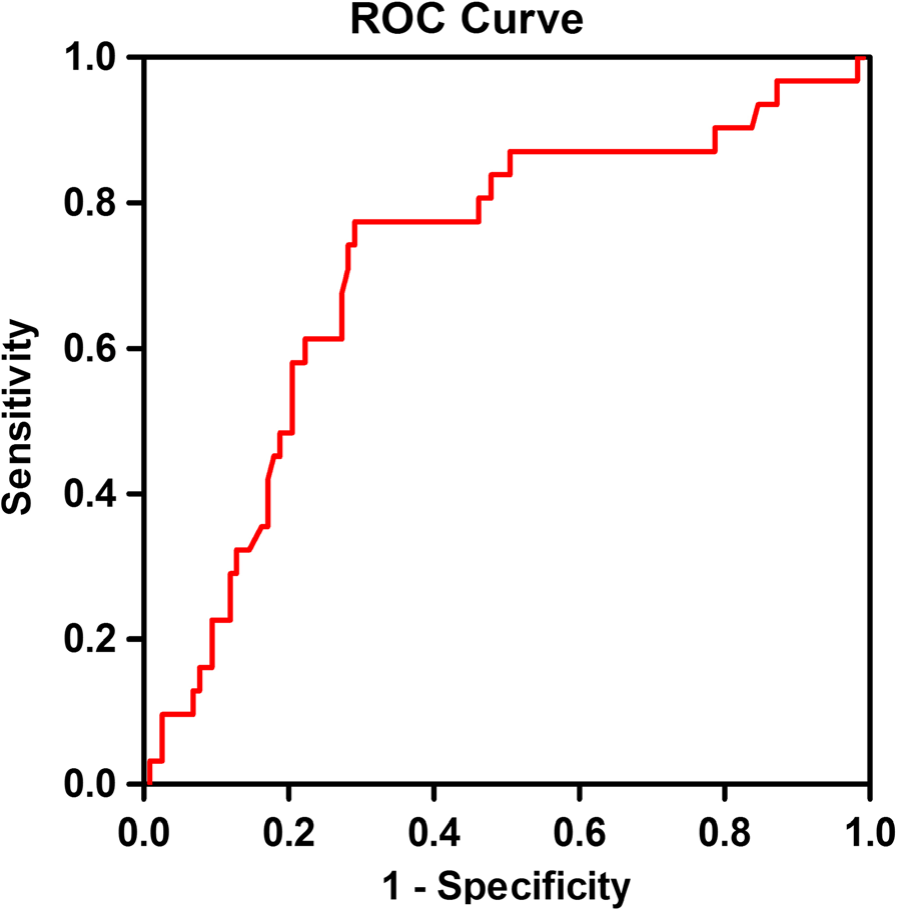
ROC curve analysis of sRAGE cut-off points in patients with PCOS

## Discussion

With an estimated prevalence of up to 10% [17], PCOS is widely accepted as the most common cause of infertility with clinical and/or biochemical signs of excess androgen secretion in the reproductive-aged women, and most of the patients have metabolic abnormalities such as hyperinsulinemia, obesity et al, implicating long-term sequelae which may affect women’s long-term health [4, 18, 19]. Therefore, searching for a new therapeutic target for PCOS is one of the highlights today. In recent years, some studies, including our research group’s preliminary work, have found that there was an elevated level of serum AGEs in PCOS women [7–9]. AGE-RAGE system is closely related to insulin resistance and hyperandrogenism, which are two core pathophysiological processes in PCOS. In addition to normal receptors, one form of AGE receptor, lacking both the cytosolic and the transmembrane domains, are called soluble RAGEs (sRAGE). They are secreted extracellularly, and can be detected in circulating blood [12, 20]. sRAGE can bind with their ligands (AGEs) in the circulation, thus preventing the adverse effects of the AGE-RAGE axis. sRAGE is then often considered a ‘good’ receptor. sRAGE levels have been shown to be down-regulated in hyperglycemia and obesity, which has been explained by the beneficial role of sRAGE and its function as decoy to capture the circulating AGEs preventing activation of RAGE signaling pathway as it still possesses the V-ligand essential for ligand binding. [13, 21, 22] Moreover, the decoy function theory is supported by an in vivo study which demonstrated that the treatment with sRAGE normalized the increases in most of the inflammatory markers in diabetic rats to those seen in age matched, non-diabetic controls. [23] But the concept of sRAGE as a decoy receptor is not unanimously supported, or at least it seems not to be complete. Because the role of sRAGE as a protective factor for vascular damage has been questioned. High levels of sRAGE have been reported associated with cardiovascular mortality in diabetic patients. Furthermore, sRAGE has been proposed as a biomarker of acute coronary syndrome, however during a very narrow time period, indicating that the time of sampling needs attention. [24] So the other soluble RAGE, esRAGE, a product of alternative splicing of the RAGE gene, also should be considered [25].

Obesity is an additional common feature of PCOS women, present in 30–75% of cases, and an aggravating factor in the cluster of clinical entities of the metabolic syndrome [26]. In previous work, it was demonstrated that serum AGEs levels are directly involved in adipogenesis [27] and production of inflammatory mediators in adipocytes, leading to complications related to obesity [28]. All these findings suggested a potential role of AGEs in obesity-related comorbid conditions. In present study, we found that serum levels of AGEs were increased, while serum levels of sRAGE were decreased along with increased BMI. Correlation analysis showed that, in line with other data [14, 29, 30], serum sRAGE levels were inversely correlated with BMI, while AGEs had a positive correlation with BMI. Multiple regression analysis found that BMI was the independent predictor factor of sRAGE, which further confirmed their findings.

Insulin resistance/hyperinsulinemia was identified as a significant contributor and possible mediator of the underlying pathophysiology in PCOS, proximately 50–70% of women with PCOS have some degree of insulin resistance [20]. In vitro studies had shown that AGEs are implicated in the pathogenesis of insulin resistance [31]. In this study, there was no correlation between AGEs and increased HOMA-IR in reproductive women with PCOS, which was consistent with Diamanti-Kandarakis E et al. research. They found that serum AGEs elevated in PCOS independently of the presence of IR, although they use the quantitative insulin sensitivity check index (QUICKI) instead of HOMA-IR represents the IR. On the other hand, earlier studies showed that serum AGEs are positively correlated with insulin and HOMA-IR in women with PCOS, as long as without hyperglycemia [12]. The hyperglycemia might account for their observation results. We found that there was an inverse relationship between sRAGE and HOMA-IR, which was accordance with the findings from Basta G et al. study [13]. The study showed that serum sRAGE concentrations were downregulated in insulin resistance status.

Hyperandrogenism is another significant contributor in the pathogenesis of PCOS. In the present study, there was no difference in AGEs with increased total testosterone levels and FAI in reproductive-aged women with PCOS. In contrast, there was an inverse relationship between sRAGE and FAI, which might be contributed to the high level of insulin inhibited liver SHBG production, and then increased FAI. sRAGE acts as a decoy receptor, which had been proved by numerous studies, and prevents the development of vascular complications [32, 33]. The decreased sRAGE levels in obese PCOS patients with more severe insulin resistance states and higher FAI could be explained by reasons below: 1) sRAGE secretion was decreased; 2) sRAGE was used more by its binding to AGEs, which were higher in these subgroup patients.

AGEs can be produced both endogenously and exogenously. The endogenous AGEs are usually formed slowly under physiological conditions, which are often relate to elevated blood glucose levels. Exogenous sources of AGEs are diet and smoking. Foods rich in protein and fat with extensive cooking, baking, grilling and frying dramatically increased AGE generation [34, 35]. Tantalaki E et al. [36] found that serum levels of AGEs, testosterone, and HOMA-IR index in high AGEs diet group were significantly higher compared to low AGEs diet. These results indicated that overexposure to exogenous AGEs may exacerbate the metabolic and hormonal disorders. Animal experiment also showed that high AGEs feed rats demonstrated higher levels of fasting glucose, insulin, testosterone, and serum AGEs than control and low AGEs-fed rats. Furthermore, the high AGEs diet group showed increased AGEs localization in the theca cells, and increased RAGE expression in granulosa cells compared to control and low AGEs-fed rats. The metabolic and hormonal alterations in conjunction with the increased dietary glycotoxins deposition in ovarian tissues in high AGEs-fed animals compared to the controls suggest an impact of environmental factors on ovarian tissue [37]. Recently, Vazzana N et al. [38] found that sRAGE significantly increases following diet-induced weight loss in obese women, and Brix JM et al. [39] also found a significant increase of sRAGE levels after a dramatic weight loss induced by bariatric surgery in adults with morbid obesity. These finding indicated that low AGEs intake, lifestyle changes along with weight loss to increase the level of sRAGE, which may effectively reduce AGE-RAGE axis’ adverse effect in PCOS patients.

Fujii EY et al. [40] found sRAGE concentrations in blood were positively correlated with the concentrations in follicular fluid (FF). Thus, the concentrations of sRAGE in blood might be able to serve as a useful biologic marker of the follicular environment. In our study, ROC curve analysis determined an optimal sRAGE cut-off point, 704.097 mmol/L in this study, for the presence of insulin resistance, reminding the requirements of intervention AGEs/sRAGE system. Another important question should be considered is whether ovary sRAGE could be biologic markers of the oocyte environment, egg quality, and outcome of fertilization, which may be more crucial in reproductive system.

## Conclusion

Taken together, our study suggested that in reproductive-aged PCOS patients, BMI was an independent risk predictive factor of AGEs and sRAGE levels. Compared with AGEs, the variation of serum sRAGE levels was more closely related to insulin resistance and hyperandrogenism (FAI), two major pathological manifestations of PCOS. AGEs/sRAGE system may be a promising intervention target in the women with PCOS, especially in obese patients, although the role of sRAGE needs be further clarified.

## Abbreviations

AGEs: Advanced glycation end-products
BMI: Body mass index
DM: Diabetes mellitus
FAI: Free androgen index
FF: Follicular fluid
FINS: Fasting insulin
FPG: Fasting plasma glucose
HOMA-IR: Homeostatic model assessment-insulin resistance
IGT: Impaired glucose tolerance
IR: Insulin resistance
PCOS: Polycystic ovary syndrome
QUICKI: Quantitative insulin sensitivity check index
SHBG: Sex-hormone-binding globulin
sRAGE: Soluble receptor of advanced glycation end-products
T2DM: Type 2 diabetes mellitus
TT: Total testosterone
WC: Waist circumference
